# Galcanezumab reduces trigeminal nociception and is effective in preclinical models of migraine and trigeminal autonomic cephalalgias

**DOI:** 10.1111/head.15006

**Published:** 2025-07-21

**Authors:** Marta Vila‐Pueyo, Peter J. Goadsby, Kirk W. Johnson, Philip R. Holland

**Affiliations:** ^1^ Headache Group, Wolfson Sensory Pain and Regeneration Centre Institute of Psychiatry, Psychology, and Neuroscience, King's College London London UK; ^2^ Eli Lilly and Company, Lilly Corporate Center Indianapolis Indiana USA

**Keywords:** cluster headache, galcanezumab, migraine, trigeminal nociceptive processing, trigeminovascular system

## Abstract

**Objectives/Background:**

This study was undertaken to assess the therapeutic efficacy of galcanezumab in preclinical models of migraine and cluster headache and to determine potential shared trigeminovascular mechanisms of action. Galcanezumab is a humanized monoclonal antibody that binds to the neuropeptide calcitonin gene‐related peptide, preventing its biological activity. It has been approved as a preventive treatment for both episodic and chronic migraine and episodic cluster headache, the most common trigeminal autonomic cephalalgia.

**Methods:**

Trigeminovascular and trigeminal–autonomic reflex activation was evoked via electrical stimulation of the dura mater or superior salivatory nucleus (SSN), respectively. Evoked responses were recorded in the spinal trigeminal nucleus along with ongoing spontaneous neuronal and cutaneous noxious‐evoked and non‐noxious‐evoked neuronal activity. Rats received either galcanezumab or human control IgG, and responses were compared between groups.

**Results:**

Galcanezumab robustly reduced spontaneous (maximum decrease in dural‐evoked: 73% [±3.5] at 4 h 30 min [*p* = 0.002]; in SSN‐evoked: 67% [±10.7] at 4 h [*p* = 0.01]) and cutaneous non‐noxious‐evoked (maximum decrease in dural‐evoked: 50% [±5.7], *p* = 0.004; in SSN‐evoked: 47% [±10.5], *p* = 0.005, at the last recording time point) neuronal activation in the trigeminocervical complex, highlighting a general inhibition of trigeminal sensory processing. Furthermore, it significantly inhibited cutaneous noxious‐evoked (maximum decrease in dural‐evoked: 38% [±5.2], *p* = 0.005; in SSN‐evoked: 34% [±7.6], *p* = 0.005, at the last recording time point), durovascular‐evoked (maximum decrease 48% [±6] at the last recording time point, *p* = 0.001), and SSN‐evoked responses (maximum decrease: 32% [±2.6] at 4 h, *p* < 0.001), demonstrating a clear reduction of trigeminal nociception, independent of the mode of activation. Galcanezumab did not have any effect on the mean arterial blood pressure.

**Conclusion:**

Galcanezumab likely acts via a shared trigeminovascular mechanism to dampen noxious and nonnoxious sensory stimuli in preclinical models of migraine and trigeminal autonomic cephalalgias. This further supports the clinical efficacy of galcanezumab for migraine and cluster headache, while demonstrating general inhibition that may be of relevance to other facial pain conditions.

## Plain Language Summary

Galcanezumab is indicated in adults for the preventive treatment of migraine and the treatment of episodic cluster headache, but the potential shared mechanisms of action in these two conditions are not clear. We tested the efficacy of galcanezumab in preclinical (rodent) models, commonly used to model migraine and cluster headache, respectively, to explore differential effects. We found that galcanezumab inhibited pain processing in both models, which suggests a general inhibition of head pain, supporting its clinical use in migraine and cluster headache and suggesting broader potential benefits for other orofacial pain/headache conditions.

**Abbreviations**
CGRP: calcitonin gene‐related peptide
SSN: superior salivatory nucleus
TAC: trigeminal autonomic cephalalgia
TCC: trigeminocervical complex
WDR: wide dynamic range

## INTRODUCTION

Galcanezumab is a humanized monoclonal antibody that binds to the neuropeptide calcitonin gene‐related peptide (CGRP), inhibiting its biological activity.^1^ Clinical studies have demonstrated its efficacy as a preventive treatment for episodic and chronic migraine and for treatment of episodic cluster headache, leading to its regulatory approval to treat both conditions, although for cluster headache this has only been approved in specific countries. Galcanezumab is thought to exert its effect in the trigeminovascular system, although its specific site of action, and of the other anti‐CGRP monoclonal antibodies, is a matter of debate due to its considered low penetrance into the central nervous system.^2^, ^3^ However, recent data have identified clear central effects of anti‐CGRP monoclonal antibodies^4^ and also passage into the cerebrospinal fluid.^5^

Migraine is a complex neurological disease characterized by the presence of attacks of unilateral, throbbing head pain accompanied by hypersensitivity to movement and visual, auditory, and other inputs.^6^ The pathophysiology of migraine‐related headache involves the activation and sensitization of the trigeminovascular pathway along with the release of CGRP, as well as altered activity and functional connectivity of key brainstem and diencephalic nuclei.^7^, ^8^, ^9^ Traditional migraine preventive treatments are nonspecific, were commonly adopted from other indications, and have several limitations, such as poor effectiveness, tolerability, and adherence.^10^ Several current and emerging migraine preventives are disease‐specific, target CGRP or its receptor, and include monoclonal antibodies and small molecule antagonists (gepants), having more favorable side effect profiles.^11^

Cluster headache is characterized by strictly unilateral headache attacks lasting 15–180 min and occurring up to eight times per day, accompanied by at least one autonomic feature ipsilateral to the pain (such as lacrimation, conjunctival injection, or rhinorrhea) and/or a sense of agitation.^6^ The pathophysiology of cluster headache likely involves the interaction of three key structures: the trigeminovascular system, the parasympathetic nerve fibers innervating the head (trigeminal–autonomic reflex), and the hypothalamus.^12^ Current preventive treatments for cluster headache are nonspecific and include mainly verapamil, lithium, melatonin, and topiramate, which are often associated with multiple adverse effects and with low and delayed efficacy.^13^, ^14^ In 2019, the use of galcanezumab as a preventive treatment option for episodic cluster headache was approved by the US Food and Drug Administration, after its effectiveness and lack of adverse events were shown in a clinical trial.^15^

Since the development of monoclonal antibodies targeting CGRP signaling, a great effort has been made to understand their mechanisms and site of action. To assess this, in the present study we analyzed the effects of intravenous galcanezumab in vivo on spontaneous, noxious‐evoked, and non‐noxious‐evoked activity of the trigeminovascular system. Moreover, we analyzed its effects in vivo in the trigeminovascular system in a validated electrophysiological model of migraine‐related trigeminal nociception,^16^ namely dural‐evoked activation of the trigeminovascular system, and in a validated model of trigeminal autonomic cephalalgias (TACs), which includes cluster headache, namely superior salivatory nucleus (SSN)‐evoked activation of the trigeminovascular system,^17^ to determine potential shared trigeminovascular mechanisms of action in these conditions.

## METHODS

### In vivo experiments

All experiments were conducted under UK Home Office Animals (Scientific Procedures) Act 1986 following approval by the local animal welfare and ethical review body and in compliance with the ARRIVE guidelines.^18^ Male Sprague Dawley rats (*n* = 32, 250–350 g, Charles River, UK) were maintained and group‐housed under standard conditions (12‐h light–dark cycle; lights on at 7:00 a.m.) with food and water freely available. The decision to use male rats only was based both on the absence of differences between male and female rats in previous studies using anti‐CGRP monoclonal antibodies,^19^ our unpublished work demonstrating estrous cycle stage‐specific alterations in the preclinical model of migraine^20^ ensuring reduced animal usage in the absence of the estrous cycle, and the known male prevalence of cluster headache in humans. All animals were randomly assigned to experimental groups (*n* = 8) based on appropriate sample size calculations performed with data from in‐house studies and an estimated effect size = 20–40%, probability of type I error = 0.05, and power = 80%, calculated using G*power software version 3.1.9.7 (Universität Düsseldorf, Düsseldorf, Germany). In each animal, only one experiment was conducted, and all analyses were therefore performed blindly on eight biological replicates.

### Surgical procedures

**FIGURE 1:**
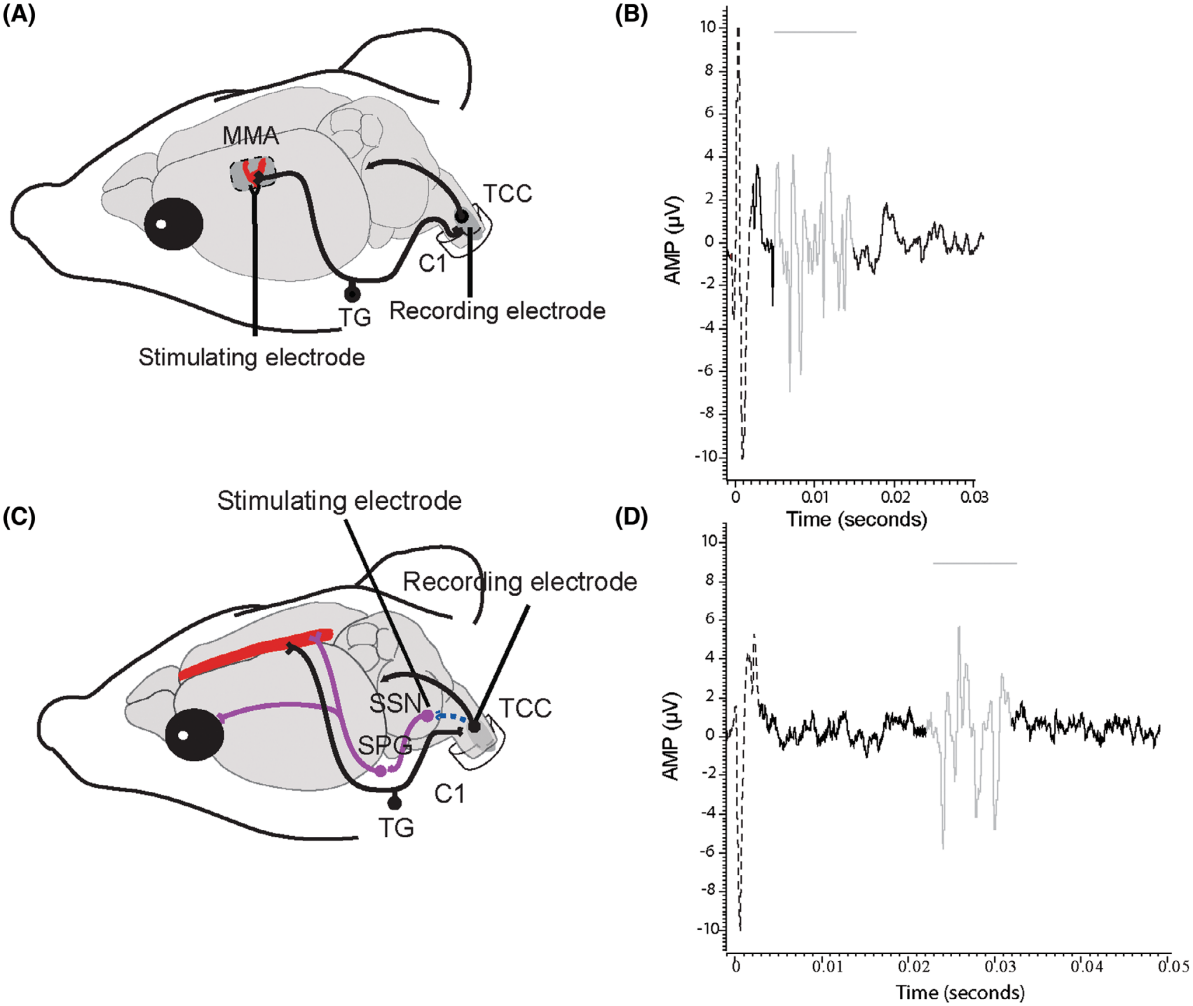
Schematic representation of the preclinical models used in the study. (A) Preclinical model of migraine‐related trigeminal nociception. A stimulating electrode is placed over the middle meningeal artery (MMA) to induce nociceptive activation of trigeminal afferents that signal via the trigeminal ganglion (TG) and innervate the dural vasculature, while projecting centrally to the trigeminocervical complex (TCC). A recording electrode placed in the TCC records nociceptive durovascular‐evoked responses. (B) An example dural‐evoked response showing neuronal activation (in gray) to durovascular stimulation (dotted line). (C) Preclinical model of trigeminal autonomic cephalalgias. A stimulating electrode is placed in the superior salivatory nucleus (SSN) to induce activation of the parasympathetic outflow via the sphenopalatine ganglion (SPG) to the cranial vasculature. This activation leads to the presence of cranial autonomic features and rebound activation of trigeminal sensory afferents, recorded in the TCC. (D) An example evoked response showing neuronal activation (in gray) to SSN stimulation (dotted line). AMP, amplitude. [Colour figure can be viewed at wileyonlinelibrary.com]

Surgical procedures agree with our previously published studies.^21^ In brief, to model durovascular‐evoked activation of the trigeminocervical complex (TCC), the parietal bone was thinned to expose the dura matter surrounding the middle meningeal artery. A bipolar stimulating electrode was placed across the middle meningeal artery to stimulate trigeminal perivascular afferents (Figure 1A,B). Next, part of the first cervical vertebrae was removed, and a tungsten recording electrode was positioned in the TCC to record the activity of wide dynamic range (WDR) neurons responding to durovascular (8–15 V, 0.5 Hz, 0.3–0.5 ms, 20 square wave electrical pulses) and periorbital (nonnoxious brush and noxious pinch) stimulation. Animals were then rested for 30 min prior to commencing, and Aδ‐fibers were identified as previously shown.^8^

To model SSN‐evoked activation of the TCC, the occipital bone was drilled and a concentric bipolar electrode was stereotaxically positioned in the SSN (9.8 mm posterior, 2.1 mm lateral, and 9.5 mm below bregma^22^; Figure 1C,D).^16^, ^17^ In agreement with the above, a tungsten recording electrode was used to record WDR neurons in the TCC activated by SSN electrical stimulation (40–65 μA, 0.5 Hz, 150–500 μs, 20 square wave electrical pulses) and with receptive fields in the ophthalmic dermatome. The responses of neurons with a latency of 13–40 ms were followed (Figure 1D). We did not assess SSN lacrimal flow responses, as these are less stable over the prolonged recording times required for assessing the efficacy of monoclonal antibodies. Electrode placement was confirmed via electrothermolytic lesion in the TCC/SSN.

In both models, cutaneous receptive fields were assessed during the whole experiment for both nonnoxious (gentle brush) and noxious (pinch) inputs. All responses were followed for 4 h 30 min after drug administration.

### Materials

Galcanezumab or human control IgG (provided by Eli Lilly & Co, Indianapolis, IN, USA, as part of an industrially funded research grant) were dissolved in physiological saline before the start of each experiment and were administered at 10 mg·kg^−1^ as an intravenous injection (volume = 0.3 mL).

### Data and statistical analysis

The data and statistical analysis were performed using SPSS 25.0 software (IBM, Armonk, NY, USA). Graphs were plotted using SigmaPlot 15.0 (Systat Software, San Jose, CA, USA) or Prism 10 (GraphPad Software, San Diego, CA, USA). All studies were designed to generate equally sized groups, and experimental animals were randomly assigned to treatment groups before being analyzed blind to the experimental group. All statistical analyses were conducted on group sizes of *n* = 8 rats, and no animals were excluded. However, two animals from the SSN‐evoked model (one from the galcanezumab and the other from the vehicle group) were only followed for 4 h instead of 4 h 30 min, hence two later timepoints of these animals are missing.

Data collected for Aδ‐fibers represent the raw data for the number of cells firing over a 10‐ms period in the region 6–17 ms (for dural‐evoked) or 13–22 ms (for SSN‐evoked) poststimulation over 20 sweeps. Spontaneous activity was measured as spikes per second (Hz) and consisted of a 500‐s time window preceding the dural stimulations using peristimulus histograms. Cutaneous nonnoxious and noxious receptive field data were measured as spikes per second (Hz) using peristimulus histograms. Raw data were first analyzed to check whether there were differences in baseline recordings between both groups (see summary of results in Table 1). If no differences were found, we proceeded to analyze raw data to assess if there was a significant effect over time using an analysis of variance for repeated measures with Bonferroni post hoc correction for multiple comparisons. If Mauchly test of sphericity was violated, appropriate corrections to the degrees of freedom were made according to Greenhouse–Geisser. If the analysis of variance for repeated measures was significant, we proceeded to conduct post hoc analyses with independent samples t‐tests. For post hoc analyses, we compared the effect of galcanezumab versus vehicle at each time point after drug administration. Data were plotted as percentage of the mean of the three baselines (normalized data). Two‐tailed statistical significance was set at *p* < 0.05. For graphical purposes, data are expressed as mean ± SEM.

**TABLE 1:** Summary of the statistical tests of the baseline responses comparison of all the recordings performed in the study.

Recording	Baseline responses comparison
Dural‐evoked model	
Spontaneous neuronal trigeminal activity	*t*_14_ = 2.924, *p* = 0.362
Cutaneous non‐noxious‐evoked trigeminal activation	*t*_14_ = −0.511, *p* = 0.618
Cutaneous noxious‐evoked trigeminal activation	*t*_14_ = −0.070, *p* = 0.945
Dural‐evoked trigeminal activation	*t*_14_ = 2.254, *p* = 0.650
Arterial blood pressure	*t*_14_ = −1.275, *p* = 0.223
SSN‐evoked model	
Spontaneous neuronal activity	*t*_14_ = −0.266, *p* = 0.794
Cutaneous non‐noxious‐evoked trigeminal activation	*t*_12_ = 1.585, *p* = 0.139
Cutaneous noxious‐evoked trigeminal activation	*t*_12_ = 1.021, *p* = 0.327
SSN‐evoked TCC activation	*t*_14_ = 1.229, *p* = 0.239
Arterial blood pressure	*t*_14_ = 1.074, *p* = 0.301

*Note*: There were no differences in the baseline responses of any of the recordings when comparing the galcanezumab with the control IgG‐treated animals.

Abbreviations: SSN, superior salivatory nucleus; TCC, trigeminocervical complex.

## RESULTS

### Durovascular‐evoked and SSN‐evoked activation of the TCC

Recordings were made from a total of 16 neuronal clusters in 16 rats for both activation methods. Extracellular recordings in the TCC were made from durovascular‐evoked or SSN‐evoked WDR neurons with cutaneous receptive fields in the ophthalmic dermatome. Evoked neuronal responses had an average latency of 6–17 ms (Figure 1B) or 13–22 ms (Figure 1D) for durovascular and SSN stimulation, respectively, and were classified as Aδ‐fibers. Neurons were located between lamina II‐V of the dorsal horn at the level of the cervicomedullary junction, at an average depth of 578 ± 44 μm and 337 ± 13 um, for durovascular and SSN‐evoked responses, respectively. Raw data have been summarized in Table 2.

**TABLE 2:** Summary of the raw data at baseline and last time point for each group and each recording.

Recording	Treatment	Raw data at baseline, mean ± SD	Raw data at last time point, mean ± SD
Dural‐evoked model			
Spontaneous neuronal activity	Control	29.75 ± 5.78 (Hz)	21.13 ± 8.18 (Hz)
	Galcanezumab	27.17 ± 4.24 (Hz)	7.25 ± 2.66 (Hz)
Cutaneous non‐noxious‐evoked trigeminal activation	Control	240 ± 45.24 (No. cells)	244.63 ± 79.78 (No. cells)
	Galcanezumab	229.38 ± 41.50 (No. cells)	119.13 ± 62.62 (No. cells)
Cutaneous noxious‐evoked trigeminal activation	Control	432.75 ± 119.06 (No. cells)	409.75 ± 95.35 (No. cells)
	Galcanezumab	429 ± 69.89 (No. cells)	266.88 ± 78.07 (No. cells)
Dural‐evoked trigeminal activation	Control	104.04 ± 4.96 (No. cells)	92.5 ± 15.84 (No. cells)
	Galcanezumab	109.21 ± 4.17 (No. cells)	57.25 ± 18.17 (No. cells)
Arterial blood pressure	Control	105.21 ± 9.72 (mmHg)	94.5 ± 8.05 (mmHg)
	Galcanezumab	100.42 ± 4.29 (mmHg)	92.75 ± 6.43 (mmHg)
SSN‐evoked model			
Spontaneous neuronal activity	Control	18.79 ± 4.37	20.57 ± 4.24
	Galcanezumab	18.04 ± 6.67	6.79 ± 5.52
Cutaneous non‐noxious‐evoked trigeminal activation	Control	213.81 ± 34.17	201 ± 78.29
	Galcanezumab	255.76 ± 61.11	131.14 ± 67.39
Cutaneous noxious‐evoked trigeminal activation	Control	358.13 ± 97.43	344.57 ± 73.35
	Galcanezumab	410.43 ± 94.14	266.86 ± 92.21
SSN‐evoked trigeminal activation	Control	102.04 ± 8.33	97.29 ± 15.33
	Galcanezumab	107.29 ± 8.75	73.14 ± 9.92
Arterial blood pressure	Control	98.46 ± 8.04	87.86 ± 9.61
	Galcanezumab	102.87 ± 8.39	90.43 ± 12.26

Abbreviation: SSN, superior salivatory nucleus.

### Galcanezumab reduces spontaneous neuronal activity in the TCC

**FIGURE 2:**
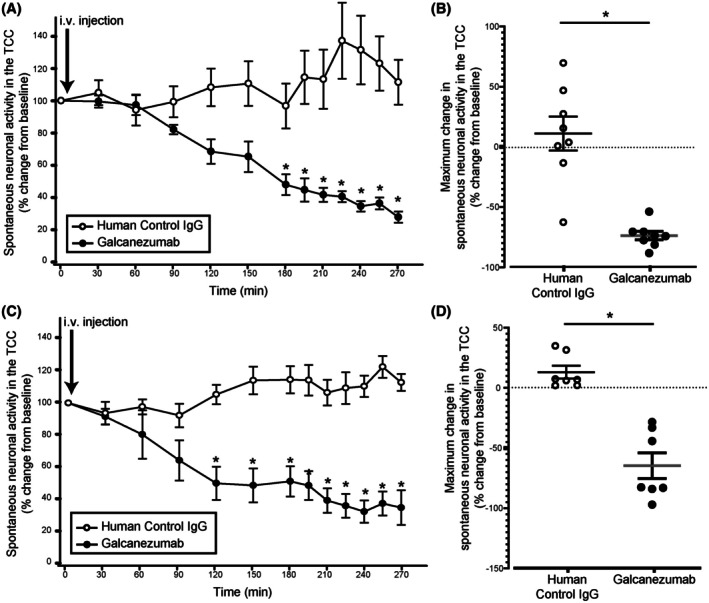
Galcanezumab reduces the spontaneous neuronal activity in the trigeminocervical complex (TCC). (A) Time course changes in the average responses of spontaneous trigeminal neuronal firing in the TCC after intravenous injection of galcanezumab or vehicle in the durovascular‐evoked groups. Galcanezumab reduced the spontaneous neuronal activity in the TCC when compared to the vehicle group (*p* < 0.001, *n* = 8/group). (B) Maximum change in the average responses of spontaneous trigeminal neuronal firing in the TCC after intravenous injection of galcanezumab compared to the vehicle in the durovascular evoked groups. (C) Time course changes in the average responses of spontaneous trigeminal neuronal firing in the TCC after intravenous injection of galcanezumab or vehicle in the SSN‐evoked groups. Galcanezumab reduced the spontaneous neuronal activity in the TCC when compared to the vehicle group (*p* = 0.007, *n* = 8/group). (D) Maximum change in the average responses of spontaneous trigeminal neuronal firing in the TCC after intravenous injection of galcanezumab compared to the vehicle in the SSN‐evoked groups. In the time course figures the points represent means, and in the maximum change figures the points represent individual values. Error bars represent SEM. i.v., intravenous. *p < 0.05.

Galcanezumab reduced spontaneous neuronal activity in the TCC when compared to control IgG‐treated animals in both the durovascular‐evoked (*F*_2.8, 39_ = 8.69, *p* < 0.001; Figure 2A,B) and SSN‐evoked (*F*_12, 168_ = 2.38, *p* = 0.007; Figure 2C,D) experiments. This inhibition was significant from 3 h, reaching a maximal response of 73% (±3.5) at 4 h 30 min (*t*_14_ = −4.56, *p* = 0.002) and 2 h, reaching a maximal response of 67% (± 10.7) at 4 h (*t*_14_ = −2.99, *p* = 0.01), for the durovascular‐evoked and SSN‐evoked experiments, respectively. Responses did not return to baseline within the recording window (Figure 2A,C).

### Galcanezumab reduces cutaneous noxious‐evoked and non‐noxious‐evoked trigeminal activation in the TCC

**FIGURE 3:**
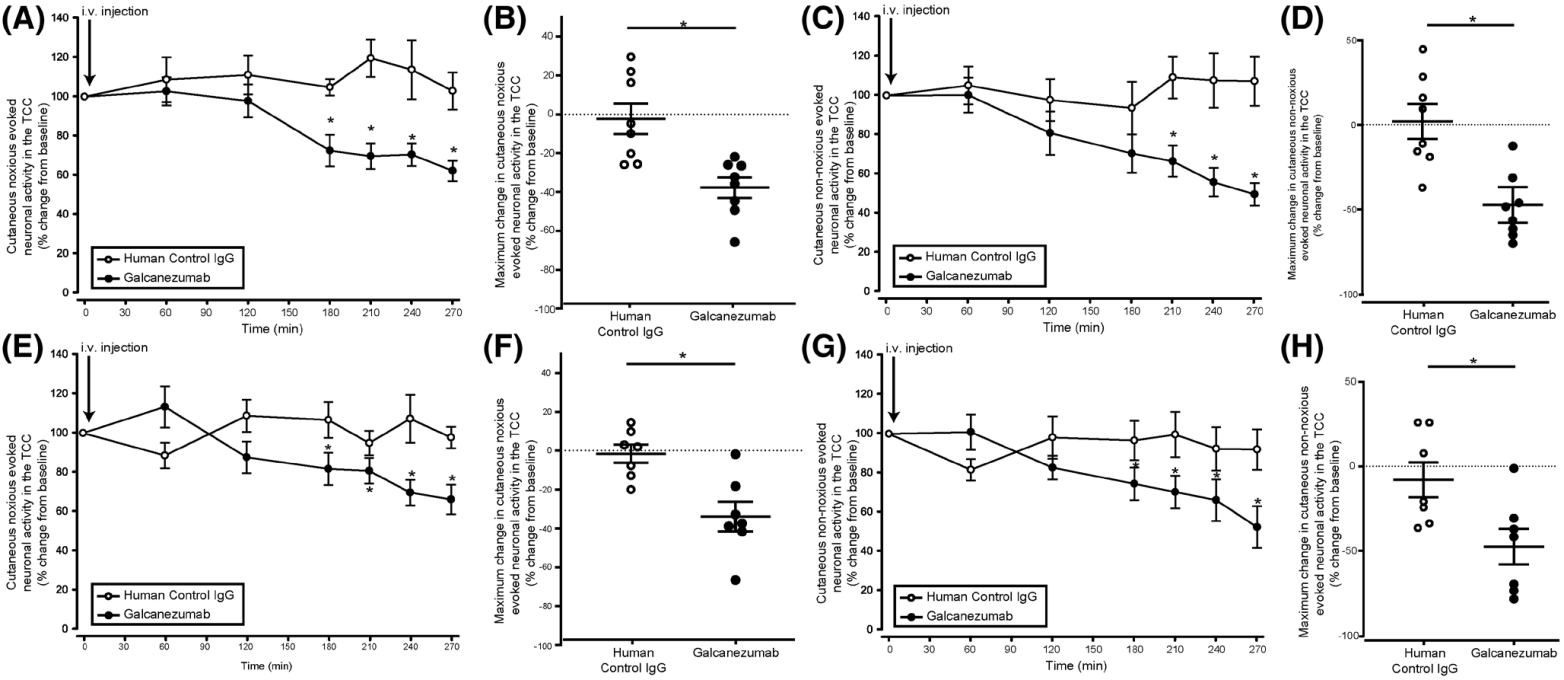
Galcanezumab reduces cutaneous noxious‐evoked and non‐noxious‐evoked trigeminal activation in the trigeminocervical complex (TCC). (A) Time course changes in the average responses of cutaneous noxious‐evoked trigeminal neuronal firing in the TCC after intravenous injection of galcanezumab or vehicle in the durovascular‐evoked groups. Galcanezumab induced a reduction of noxious‐evoked responses in the TCC when compared to the vehicle group (*p* < 0.001, *n =* 8/group). (B) Maximum change in the average responses of cutaneous noxious‐evoked trigeminal neuronal firing in the TCC after intravenous injection of galcanezumab compared to the vehicle in the durovascular‐evoked groups. (C) Time course changes in the average responses of cutaneous non‐noxious‐evoked trigeminal neuronal firing in the TCC after intravenous injection of galcanezumab or vehicle in the durovascular‐evoked groups. Galcanezumab induced a reduction of non‐noxious‐evoked responses in the TCC when compared to the vehicle group (*p* < 0.001, *n =* 8/group). (D) Maximum change in the average responses of cutaneous non‐noxious‐evoked trigeminal neuronal firing in the TCC after intravenous injection of galcanezumab compared to the vehicle in the durovascular‐evoked groups. (E) Time course changes in the average responses of cutaneous noxious‐evoked trigeminal neuronal firing in the TCC after intravenous injection of galcanezumab or vehicle in the superior salivatory nucleus (SSN)‐evoked groups. Galcanezumab induced a reduction of noxious‐evoked responses in the TCC when compared to the vehicle group (*p* = 0.019, *n =* 8/group). (F) Maximum change in the average responses of cutaneous noxious‐evoked trigeminal neuronal firing in the TCC after intravenous injection of galcanezumab compared to the vehicle in the SSN‐evoked groups. (G) Time course changes in the average responses of cutaneous non‐noxious‐evoked trigeminal neuronal firing in the TCC after intravenous injection of galcanezumab or vehicle in the SSN‐evoked groups. Galcanezumab induced a reduction of non‐noxious‐evoked responses in the TCC when compared to the vehicle group (*p* = 0.018, *n =* 8/group). (H) Maximum change in the average responses of cutaneous non‐noxious‐evoked trigeminal neuronal firing in the TCC after intravenous injection of galcanezumab compared to the vehicle in the SSN‐evoked groups. In the time course figures the points represent means, and in the maximum change figures the points represent individual values. Error bars represent SEM. i.v., intravenous. *p* < 0.05.

Galcanezumab reduced cutaneous noxious‐evoked and non‐noxious‐evoked neuronal activity in the TCC when compared to control IgG‐treated animals in both the durovascular‐evoked (*F*_6, 84_ = 5.58, *p <* 0.001; Figure 3A–D) and SSN‐evoked (*F*_6, 78_ = 2.72, *p* = 0.019; Figure 3E–H) experiments. In the durovascular model, the noxious responses were inhibited from 3 h postadministration, maximally by 38% (±5.2) at 4 h 30 min (*t*_14_ = −3.28, *p* = 0.005; Figure 3B), whereas for nonnoxious responses this effect was significant from 3 h 30 min postadministration, maximally by 50% (±5.7) at 4 h 30 min (*t*_14_ = −3.5, *p* = 0.004; Figure 3D). In the SSN‐evoked model, both noxious and nonnoxious responses were significantly reduced from 3 h postadministration, maximally by 34% (±7.6) at 4 h 30 min (*t*_14_ = 4.3, *p* = 0.005; Figure 3F) and by 47% (±10.5) at 4 h 30 min (*t*_14_ = 4.2, *p* = 0.005; Figure 3H), respectively.

### Galcanezumab reduces durovascular‐evoked and SSN‐evoked nociceptive responses in the TCC

**FIGURE 4:**
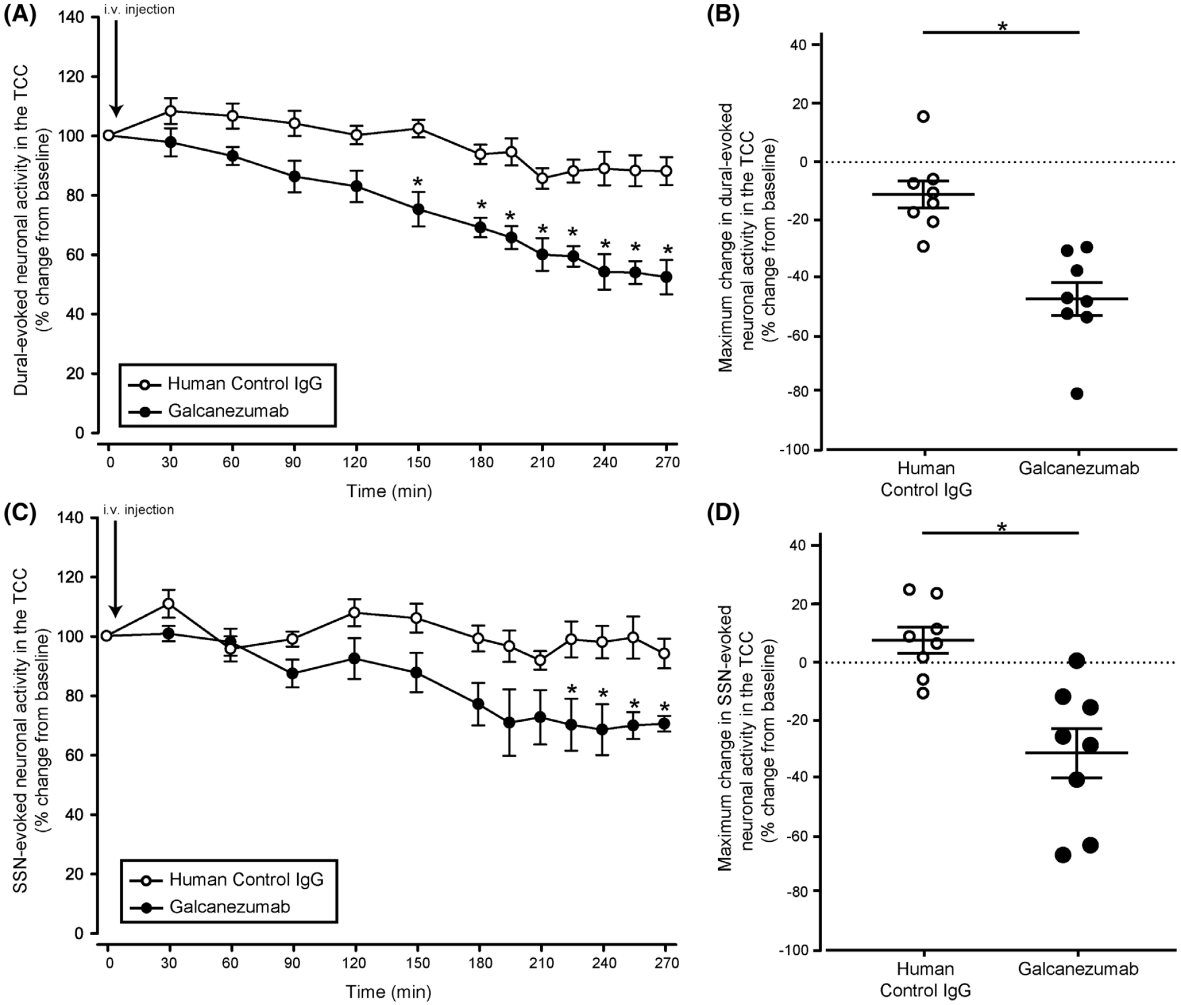
Galcanezumab reduces durovascular and superior salivatory nucleus (SSN)‐evoked nociceptive responses in the trigeminocervical complex (TCC). (A) Time course changes in the average responses of dural‐evoked Aδ‐fiber trigeminal neurons in the TCC after intravenous injection of galcanezumab or vehicle. Galcanezumab reduced the dural‐evoked neuronal activity in the TCC compared to the vehicle group (*p* < 0.001, *n* = 8/group). (B) Maximum change in the average responses of dural‐evoked Aδ‐fiber trigeminal neurons in the TCC after intravenous injection of galcanezumab compared to the vehicle group. (C) Time course changes in the average responses of SSN‐evoked trigeminal neurons in the TCC after intravenous injection of galcanezumab or vehicle. Galcanezumab reduced the SSN‐evoked neuronal activity in the TCC over time compared to vehicle‐treated rats (*p* = 0.001, *n* = 8/group). (D) Maximum change in the average responses of SSN‐evoked trigeminal neurons in the TCC after galcanezumab injection compared to vehicle group. In the time course figures the points represent means, and in the maximum change figures the points represent individual values. Error bars represent SEM. i.v., intravenous. *p* < 0.05.

Galcanezumab induced a significant reduction in durovascular‐evoked nociceptive activation in the TCC when compared to control IgG‐treated animals (*F*_5.4, 76_ = 26.47, *p* < 0.001; Figure 4A). Specifically, galcanezumab reduced the durovascular‐evoked activation of the TCC from 2 h 30 min postadministration, maximally by 48% (±6) at the last recording time point (4 h 30 min, *t*_14_ = −4.14, *p* = 0.001; Figure 4B). In agreement, galcanezumab significantly reduced SSN‐evoked nociceptive activation in the TCC when compared to vehicle‐treated animals (*F*_3.7, 48_ = 5.44, *p* = 0.001; Figure 4C). Specifically, galcanezumab reduced the SSN‐evoked activation of TCC neuronal responses from 3 h 45 min postadministration, reaching a maximum decrease of 32% (± 2.6) at 4 h (*t*_13_ = −7.17, *p* < 0.001) that did not return to baseline within the recording window (Figure 4D).

### Effect of galcanezumab on mean arterial blood pressure

**FIGURE 5:**
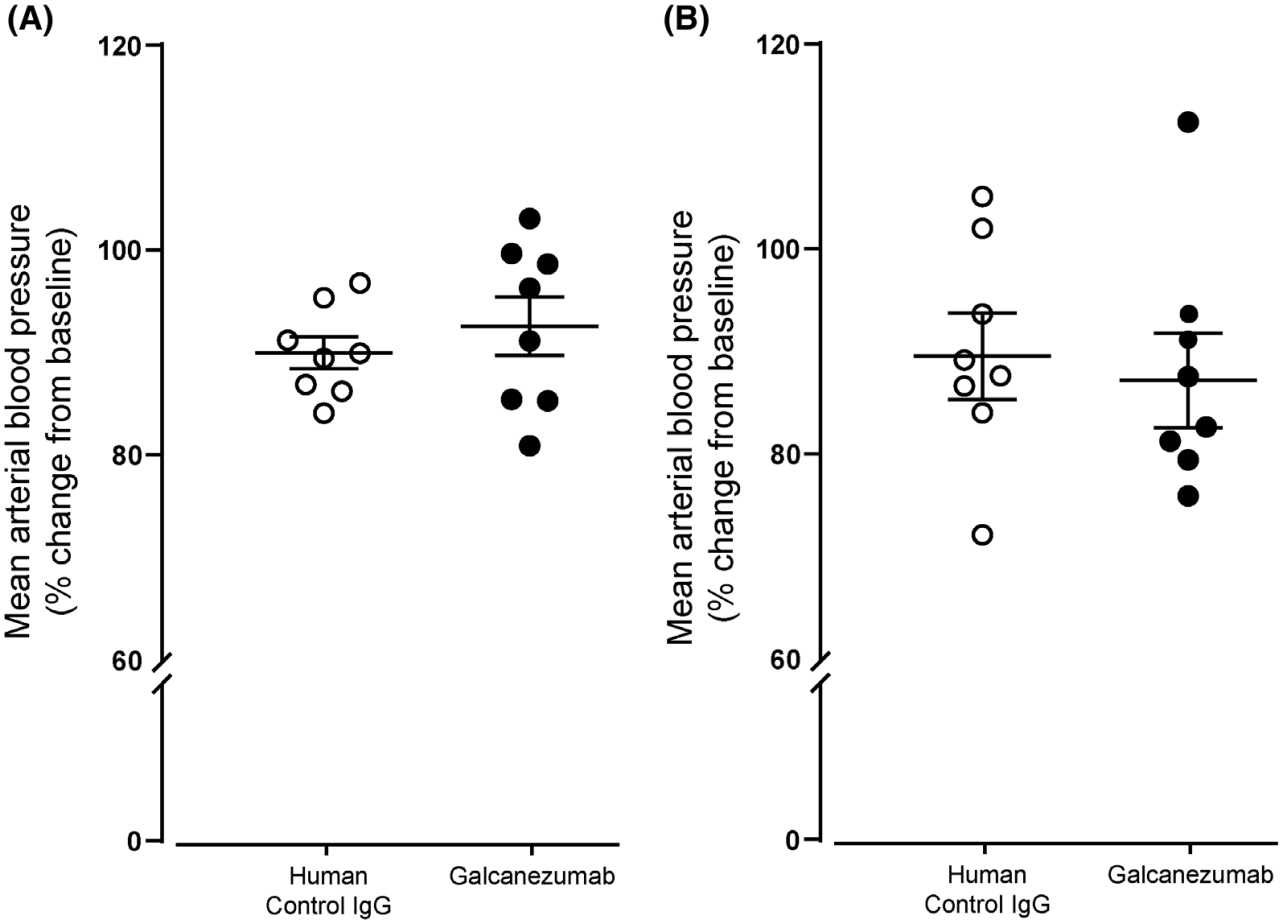
Galcanezumab does not change mean arterial blood pressure. The intravenous injection of galcanezumab did not modify the mean arterial blood pressure when compared to the human IgG control group in (A) the durovascular‐evoked group (*p* = 0.638, *n* = 8/group) or in (B) the superior salivatory nucleus‐evoked group (*p* = 0.055, *n* = 8/group). The points represent individual values, and error bars represent SEM.

Galcanezumab‐treated animals showed no change in mean arterial blood pressure in either the durovascular‐evoked (*t*_14_ = −0.48, *p* = 0.638; Figure 5A) or SSN‐evoked (*F*_1.8, 25_ = 3.36, *p* = 0.055; Figure 5B) responses when compared to control IgG‐treated animals.

## DISCUSSION

In this study, we demonstrate that galcanezumab, an anti‐CGRP humanized monoclonal antibody, inhibits general trigeminovascular activity in two distinct preclinical models designed to model migraine‐related durovascular and cluster headache‐related SSN‐evoked activation in the TCC. Specifically, galcanezumab inhibited spontaneous activity of WDR neurons and their evoked responses to both durovascular and SSN stimulation. In addition, galcanezumab inhibited both noxious and nonnoxious responses to cutaneous receptive field stimulation. Hence, our results show a strong effect of galcanezumab in decreasing neuronal firing and activation of the trigeminovascular system, recorded at the level of the TCC in naïve rats, that is independent from the specific mode of activation, in agreement with the literature.^19^

Importantly, the current results highlight differences with alternate antibodies, whereby fremanezumab inhibited only high threshold and not WDR neurons in the TCC in naïve or cortical spreading depression‐sensitized rats. Furthermore, fremanezumab had no effect on cutaneous responses, whereas in the current study galcanezumab had a modest inhibitory action. Importantly, CGRP has many broad functions in sensory processing beyond nociception from mechanical sensitivity^23^ to the central integration of threatening signals.^24^ The exact mechanisms underlying these differences in responses to two different monoclonal antibodies targeting CGRP are unclear; clinically there is little difference in terms of a reduction in mean monthly days. However, galcanezumab has demonstrated a rapid onset of within 1 week in migraine^25^ and a more rapid onset effect in medication‐overuse headache when compared to alternate monoclonal antibodies.^26^ With respect to cutaneous responses, galcanezumab has demonstrated a significant acute effect on cutaneous‐evoked responses from the ophthalmic dermatome in patients that was no longer evident following prolonged use^27^; hence, acute cutaneous responses may represent a transient impact of inhibiting CGRP and are not overall concerning, considering similar effects of several migraine preventives (e.g., flunarizine, sodium valproate, amitriptyline, and lamotrigine) in this model.^28^ Eptinezumab given intravenously in patients has demonstrated a rapid onset of effect within 2 h,^29^ in agreement with the rapid onset (3–4 h) observed in the current study following intravenous galcanezumab. Unfortunately, given the systemic administration used in this and similar studies, a direct conclusion regarding the exact site and mechanisms of action of galcanezumab is beyond the scope of the current study.

For this reason, we originally sought to explore the potential action of galcanezumab on the trigeminovascular system and trigeminal–autonomic reflex as preclinical models of migraine and cluster headache‐related responses, respectively. However, direct monitoring of the parasympathetic outflow to the face proved unstable over the 6–8‐h surgical and recording duration required, meaning that we could not assess a direct action on the cranial parasympathetic pathway. Therefore, we cannot rule out potential differential actions in the two models; however, we did observe comparable effects of galcanezumab on spontaneous neuronal activity and cutaneous‐evoked responses in the TCC, suggesting a likely shared inhibition of trigeminovascular activation as the key factor. Of note, recording evoked responses in the TCC is responsive to peripheral and central modulation, including central pharmacological effects.^30^, ^31^

Previous studies have investigated distribution of monoclonal antibodies in rats, demonstrating the highest levels in the dura mater and spleen, followed by the trigeminal ganglion, with lower levels in the hypothalamus, spinal cord, prefrontal cortex, cerebellum, and cerebrospinal fluid.^3^ This preferential distribution is supported by ex vivo experiments highlighting fluorophore‐labeled galcanezumab in the dura mater and trigeminal ganglion for at least 30 days after subcutaneous injection, with resultant reduced capsaicin‐evoked CGRP release, specifically in females.^32^

Taken altogether, the results of these studies indicate that galcanezumab exerts its main effects on the peripheral parts of the trigeminovascular system via the inhibition of CGRP signaling; however, their peripheral actions potentially modulate central networks that may contribute to the preventive effect. This is supported by clinical studies where anti‐CGRP monoclonal antibodies improve central premonitory and accompanying symptoms of the headache phase.^33^ Furthermore, galcanezumab decreases hypothalamic activation in patients, most robustly in responders^4^ and interestingly, this response was not present with antibodies that specifically target the CGRP receptor, suggesting a preferential central response to antibodies that target CGRP itself.^4^ Moreover, recent clinical data demonstrate that CGRP‐targeted antibodies can access the cerebrospinal fluid in a 1:1000 ratio compared to the plasma, and binds CGRP in the cerebrospinal fluid over a 1‐month period in humans.^5^ Given the difference between blood–brain barrier and cerebrospinal fluid–interstitial fluid transfer^34^ and the proximity of many structures implicated in migraine, including hypothalamic, periaqueductal gray, and thalamic regions with the cerebrospinal fluid, the issue of the site of action seems not entirely resolved, supported by a small but not negligible presence of galcanezumab in the cerebrospinal fluid of rodents following administration.^3^

Importantly, our study demonstrates preclinical support of the beneficial effects of galcanezumab in cluster headache. We used a preclinical model that is based on the activation of the trigeminal–autonomic reflex by stimulating the SSN and recording nociceptive activation in the TCC.^17^ The results obtained in this model show a strong, significant effect of galcanezumab in the reduction of SSN‐evoked nociceptive trigeminal TCC responses, indicating that galcanezumab likely reduces trigeminal nociceptive traffic via the TCC to decrease pain symptoms in cluster headache; however, the inclusion of only male rats excludes the study of potential sex differences, which is a limitation. We had previously shown a beneficial effect of lasmiditan,^16^ naratriptan,^17^ oxygen,^17^ and indomethacin,^17^ indicating the translatability of this model to assess the therapeutic effects of drugs for TACs, including cluster headache. Galcanezumab was approved by the US Food and Drug Administration for treatment of episodic cluster headache,^15^ and although in clinical practice, patients with chronic cluster headache have been shown to benefit from its treatment,^35^ the phase 3 randomized controlled trial on chronic cluster headache did not achieve its primary and key secondary endpoints,^36^ and additional clinical trials remain ongoing.^37^, ^38^, ^39^

Besides the migraine‐relevant effects of CGRP on nociception, it is also an important vasodilator and is thought to protect organs during cardiac and/or cerebral ischemia, which has raised concerns on the cardiovascular safety of anti‐CGRP monoclonal antibodies.^40^ Long‐term studies have shown that these drugs are safe and well‐tolerated,^41^ and our data demonstrate that galcanezumab has no immediate effect on mean arterial blood pressure.

## CONCLUSION

Taken together, our results demonstrate a significant action of galcanezumab in reducing nociceptive trigeminovascular neuronal activation at the level of the TCC in two well‐established in vivo models of primary headaches: migraine and TACs, including cluster headache. These data support the mechanism of action of galcanezumab as an antimigraine and anti‐cluster headache treatment in the trigeminovascular system.

## AUTHOR CONTRIBUTIONS

**Marta Vila‐Pueyo:** Conceptualization; data curation; formal analysis; methodology; writing – original draft; writing – review and editing. **Peter J. Goadsby:** Conceptualization; funding acquisition; writing – review and editing. **Kirk W. Johnson:** Conceptualization; writing – review and editing. **Philip R. Holland:** Conceptualization; funding acquisition; methodology; project administration; supervision; writing – review and editing.

## FUNDING INFORMATION

This study received funding support from Eli Lilly and Company.

## CONFLICT OF INTEREST STATEMENT

**Marta Vila‐Pueyo** declares no competing financial interests. **Peter J. Goadsby** reports, unrelated to the current project, over the past 36 months, grants from Celgene and Kallyope; personal fees from Eon Biopharma, AbbVie, Amgen, eNeura, CoolTech, Dr. Reddy's, Eli Lilly and Company, Epalex, Linpharma, Lundbeck, Man&Science, Novartis, Pfizer, Sanofi, Satsuma, Shiratronics, and Teva Pharmaceuticals; personal fees for advice through Gerson Lehrman Group, Guidepoint, SAI Med Partners, and Vector Metric; fees for educational materials from CME Outfitters; publishing royalties or fees from Massachusetts Medical Society, Oxford University Press, UpToDate, and Wolters Kluwer; and a patent magnetic stimulation for headache (No. WO2016090333 A1) assigned to eNeura without fee. **Philip R. Holland** reports, unrelated to the current project, honoraria for educational and advisory purposes from Allergan, Novartis, and Teva as well as research funding from Eli Lilly and Company, Amgen, and Cellgene/Bristol Myers Squibb. **Kirk W. Johnson** is a full‐time employee and stockholder of Eli Lilly and Company.

## Data Availability

The data that support the findings of this study are available from the corresponding author upon reasonable request. Some data may not be made available because of privacy or ethical restrictions.
